# Prolonged venous transit on perfusion imaging predicts discharge Cog-4 scores in anterior-circulation large-vessel occlusion stroke

**DOI:** 10.1016/j.neurot.2026.e00919

**Published:** 2026-06-06

**Authors:** Hamza Adel Salim, Andrew Cho, Derek Tsang, Dhairya A. Lakhani, Risheng Xu, Shyam Majmundar, Mona Gad, Vaibhav Vagal, Shobit Chamoli, Karthik Lalwani, Ferdinand Hui, Adam A. Dmytriw, Adrien Guenego, Kambiz Nael, Gregory W. Albers, Jeremy J. Heit, Tobias D. Faizy, Max Wintermark, Vivek Yedavalli

**Affiliations:** a Department of Radiology, Division of Neuroradiology, Johns Hopkins Medical Center, Baltimore, MD, USA; b Department of Neuroradiology, MD Anderson Medical Center, Houston, TX 77030, USA; c Department of Neuroradiology, Rockefeller Neuroscience Institute, West Virginia University, Morgantown, WV, USA; d Department of Neurology, University of Maryland Medical Center, Baltimore, MD, USA; e Renaissance School of Medicine at Stony Brook University, USA; f Department of Radiology, Armed Forces Medical College, Pune, India; g Division of Radiology, Queen's Medical Center, University of Hawaii, Hawaii, USA; h Neuroendovascular Program, Massachusetts General Hospital, Harvard University, Boston, MA, USA; i Neurovascular Centre, Department of Medical Imaging and Neurosurgery, St. Michael's Hospital, Toronto, ON, Canada; j Department of Diagnostic and Interventional Neuroradiology, Erasme University Hospital, Brussels, Belgium; k Department of Radiology & Biomedical Imaging, University of California, San Francisco, CA, USA; l Department of Interventional Neuroradiology, Stanford Medical Center, Palo Alto, CA, USA; m Department of Radiology, Neuroendovascular Program, University Medical Center Münster, Germany; n Department of Radiology, University of Texas Medical Branch, Galveston, TX, USA

**Keywords:** Acute ischemic stroke, Large-vessel occlusion, Prolonged venous transit, Perfusion imaging, Post-stroke cognitive impairment, Cog-4

## Abstract

Post-stroke cognitive impairment is common after acute ischemic stroke, yet early cognitive deficits are often underrecognized in routine care. Prolonged venous transit (PVT) on perfusion imaging reflects impaired venous drainage and has been linked to worse functional outcomes in large-vessel occlusion (LVO) stroke, but its relationship with early cognitive impairment remains unclear. In this retrospective study, we evaluated consecutive patients with anterior-circulation LVO who underwent baseline perfusion imaging and had a documented discharge Cog-4 score (derived from four NIHSS items: orientation, command following, language, and attention/neglect). PVT was defined as a Tmax delay ≥10 s within the posterior superior sagittal sinus or torcula. The primary outcome was discharge Cog-4 score. Among 253 patients, 85 (34%) had PVT. Patients with PVT had higher Cog-4 scores than those without PVT (median, 2 vs 0; P < 0.001). In multivariable linear regression adjusting for age, admission NIHSS score, occlusion laterality, and follow-up infarct volume, PVT remained independently associated with higher Cog-4 scores (β, 0.63; 95% CI, 0.07–1.2; P = 0.029). A multivariable model demonstrated good discrimination for identifying normal or minimal impairment (Cog-4 score 0–1) with an area under the curve of 0.86 (95% CI, 0.81–0.90). These findings suggest that venous outflow impairment on baseline perfusion imaging is independently associated with early cognitive dysfunction at hospital discharge and may serve as a practical imaging marker for early cognitive risk stratification in anterior-circulation LVO stroke.

## Introduction

Post-stroke cognitive impairment is common after acute ischemic stroke (AIS) and contributes substantially to disability and loss of independence. Cognitive deficits affect up to 60% of survivors within the first year after stroke [1], and approximately one-third develop dementia within five years [1]. Early cognitive decline influences functional recovery, caregiver burden, and the ability to return to premorbid roles [[1], [2], [3]]. Despite its clinical relevance, cognitive impairment is frequently underrecognized in routine stroke care, in part because early evaluations emphasize motor and language deficits rather than cognition [1].

Standardized screening instruments such as the Montreal Cognitive Assessment (MoCA) and the Mini-Mental State Examination (MMSE) provide broader assessment across cognitive domains but may be limited in the acute stroke setting by aphasia, neglect, reduced arousal, and time constraints [1,4]. In this context, the NIHSS-derived cognitive items (Cog-4), composed of orientation questions, command following, best language, and extinction/inattention, has been proposed as a pragmatic discharge-stage measure of higher cortical deficit burden that can be obtained from routinely documented NIHSS items without additional testing.^56^ In prior work, Cog-4 showed moderate discrimination for severe cognitive impairment 18 months after stroke (AUC, 0.78) [5]. Although Cog-4 is not a domain-based cognitive screen, it quantifies higher cortical neurologic deficits (language, attention/neglect, and related items) that are core contributors to functional cognitive limitations early after stroke and that often motivate subsequent formal cognitive evaluation.

Venous outflow impairment has recently been recognized as a contributor to clinical outcomes after AIS [[6], [7], [8], [9]]. Prolonged venous transit (PVT), identified on perfusion imaging as markedly delayed Tmax in the major venous sinuses, reflects impaired venous drainage and has been associated with worse neurological and functional outcomes in large-vessel occlusion stroke [[10], [11], [12], [13]]. Although cognitive impairment is well documented in conditions characterized by venous congestion, including cerebral venous sinus thrombosis and idiopathic intracranial hypertension [[14], [15], [16], [17]], the relationship between venous outflow impairment and higher cortical deficit burden after arterial ischemic stroke has not been established.

This study aimed to determine whether PVT on baseline imaging perfusion is associated with higher cortical deficit burden, as measured by the Cog-4, in patients with anterior-circulation large-vessel occlusion.

## Methods

This retrospective study was approved by the institutional review board with waiver of informed consent (JHU-IRB00269637) [[18], [19], [20], [21], [22], [23], [24], [25], [26], [27], [28], [29], [30], [31], [32]]. This study follows the Strengthening the Reporting of Observational Studies in Epidemiology checklist guidelines as an observational study.

### Population

From a prospective registry of patients with acute ischemic stroke between September 1, 2017 and September 22, 2022, we retrospectively analyzed patients meeting inclusion criteria. Inclusion criteria were: (1) confirmed anterior circulation large vessel occlusion (LVO) on baseline CT angiography (CTA) as part of a comprehensive CT evaluation (noncontrast CT, CTA, and CT perfusion [CTP]); and (2) available Cognitive Assessment for Stroke Patients (Cog-4) score at hospital discharge. Patients without LVO etiology or without available perfusion or cognitive data were excluded.

### Clinical data collection

Baseline clinical data were ascertained by certified neurologists or nurse practitioners at the time of the clinical encounter and were retrospectively extracted from the electronic medical record. These parameters included demographics, risk factors for acute ischemic stroke (including diabetes, hypertension, coronary artery disease, and atrial fibrillation), prior stroke history, antiplatelet or anticoagulant use, tobacco use, alcohol use, admission glucose, premorbid mRS score and admission National Institutes of Health Stroke Scale (NIHSS) score.

### Clinical outcomes assessment

The primary outcome was cortical deficit burden at hospital discharge, measured by the Cog-4 score. The Cog-4 was derived from four NIHSS items (level of consciousness questions, level of consciousness commands, best language, and extinction/inattention) summed to yield a total score of 0–9, with higher scores indicating greater cortical deficit burden. The Cog-4 was calculated from the last documented NIHSS assessment before discharge. We used Cog-4 as a pragmatic discharge-stage measure of higher cortical dysfunction that reflects early cognitive–behavioral limitations in routine stroke workflows.

### Interventional data collection

Reperfusion treatment followed institutional protocols. The decision to administer intravenous thrombolysis (IVT) and/or perform mechanical thrombectomy (MT) was made by a multidisciplinary stroke team. For MT, device selection (aspiration catheter, stent retriever, or combined approach) and number of passes were at the discretion of the neurointerventionalist. Time intervals from last-known-well to hospital arrival and from arrival to reperfusion were recorded. Final angiographic reperfusion was graded using the modified Thrombolysis in Cerebral Infarction (mTICI) scale by the treating neurointerventionalist.

### Imaging data collection

Baseline imaging included noncontrast CT, CTA, and CTP performed on a Siemens Somatom Force scanner (Siemens Healthineers, Erlangen, Germany). CTP parameters were: 70 kVp, 200 effective mAs, rotation time 0.25 s, total acquisition time 60 s, collimation 48 × 1.2 mm, pitch 0.7, and 4D coverage of 114 mm × 1.5 s. CTP datasets were postprocessed with RAPID software (iSchemaView, Menlo Park, CA) to generate perfusion maps of relative cerebral blood flow (rCBF), cerebral blood volume (CBV), and time-to-maximum (Tmax).

Two board-certified neuroradiologists, to blinded clinical data, independently reviewed baseline and follow-up imaging. Alberta Stroke Program Early CT Scores (ASPECTS) were derived from pretreatment noncontrast CT. Site of occlusion (internal carotid artery, M1, or M2 segment) and laterality were determined on CTA. Hemorrhagic transformation was classified according to European Cooperative Acute Stroke Study (ECASS II) criteria on follow-up CT or susceptibility-weighted MRI performed within 48 h [33,34]. Discrepancies were resolved by consensus.

### PVT assessment

**Fig. 1:**
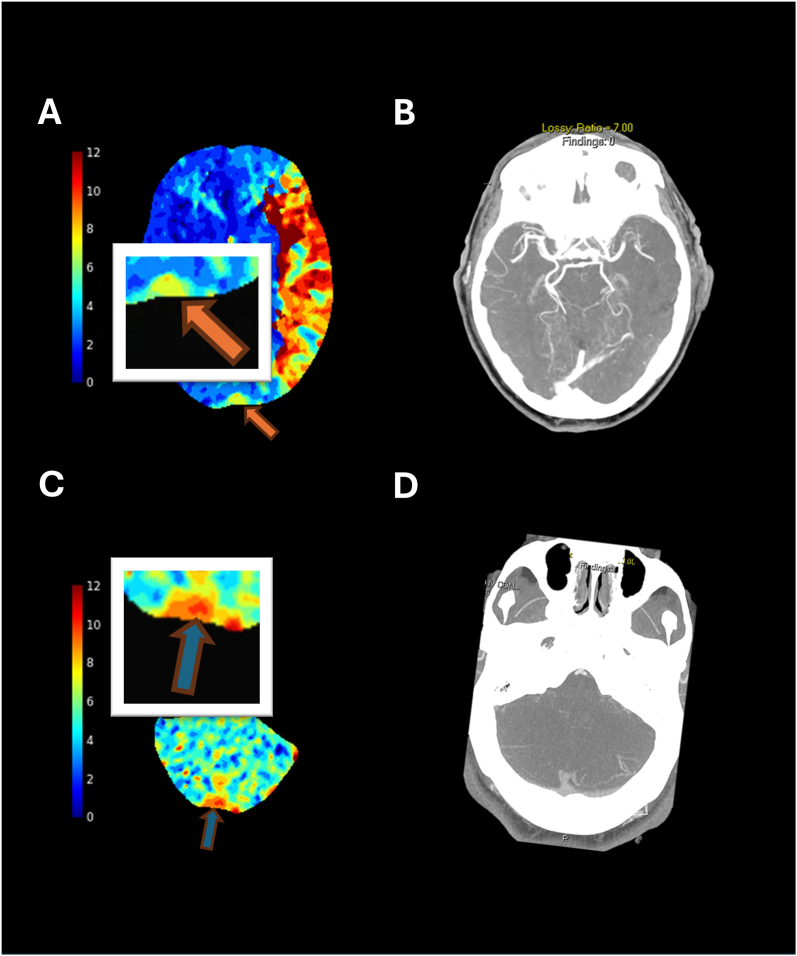
Association between venous outflow status and discharge higher cortical dysfunction (Cog-4) in two patients with anterior circulation large vessel occlusion. (A, B) Images in a 75-year-old man who presented with right facial droop and extremity weakness (admission NIHSS score, 14). (A) Baseline CT perfusion Tmax map shows normal venous transit within the torcula and superior sagittal sinus (orange arrow). (B) CT angiogram source image shows corresponding anatomy. After mechanical thrombectomy (mTICI grade 2b achieved after one pass), the patient demonstrated excellent cognitive recovery at discharge (Cog-4 score, 0). (C, D) Images in a 66-year-old woman who presented with left middle cerebral artery syndrome and high stroke severity (admission NIHSS score, 17). (C) Baseline CT perfusion Tmax map reveals prolonged venous transit, characterized by marked delay in the torcula (blue arrow). (D) CT angiogram source image shows corresponding anatomy. Despite undergoing successful mechanical thrombectomy (mTICI grade 2b after one pass), the patient exhibited significant cognitive impairment at discharge (Cog-4 score, 5).

Qualitative assessment of venous outflow was performed using Tmax perfusion maps. The posterior superior sagittal sinus (SSS) at the level of the occipital horn and the torcula were examined to capture both superficial and deep venous drainage patterns. PVT+ was defined as Tmax delay ≥10 s within either region when referenced to the color timing key. Assessments were performed independently by a fellowship-trained neuroradiologist and a neuroradiology fellow, blinded to outcomes, with disagreements resolved by consensus review. Inter-rater agreement for PVT classification was quantified with Cohen's kappa (k = 0.79, 95% CI: 0.69–0.88) [35]. Representative cases are shown in Fig. 1.

### Statistical analysis

The primary objective was to determine the association between PVT status and cortical deficit burden at discharge (Cog-4 score). Continuous variables were expressed as medians with interquartile ranges (IQRs) and compared using the Wilcoxon rank-sum test, while categorical variables were summarized as counts and percentages and compared using χ^2^ or Fisher exact tests, as appropriate.

Univariable linear regression was performed to identify variables associated with discharge Cog-4 score at a significance threshold of p < 0.10. Variables meeting this criterion, along with clinically relevant covariates, were entered into a multivariable linear regression model using stepwise backward elimination. Results were reported as β coefficients with 95% confidence intervals (CIs), and statistical significance was defined as a two-sided p < 0.05.

To assess the discriminatory performance of PVT and the final multivariable model in identifying patients with normal or minimal higher cortical deficits (Cog-4 score 0–1), receiver operating characteristic (ROC) curve analysis was performed. The area under the curve (AUC) with 95% CI was calculated for each predictor. As a sensitivity analysis, we additionally performed ordinal logistic regression to evaluate the association between candidate variables and discharge Cog-4 score, given the ordinal nature of the outcome. All analyses were conducted using R software (version 4.2.2; R Foundation for Statistical Computing, Vienna, Austria).

## Results

### Study population

A total of 253 patients with anterior-circulation large-vessel occlusion who underwent baseline perfusion imaging and had a discharge Cog-4 score were included in the analysis. PVT was present in 85 patients (34%) and absent in 168 (66%).

### Baseline characteristics

Patients with PVT were older than those without PVT (median age, 70 years [IQR, 63 to 81] vs. 66 years [IQR, 55 to 77]; P = 0.003) and more likely to be male (56% vs. 39%; P = 0.009) (Table 1). Admission stroke severity was higher in the PVT group (median NIHSS score, 17 [IQR, 13 to 20] vs. 13 [IQR, 9 to 20]; P = 0.003). Baseline infarct core volume (rCBF <30% volume) did not differ significantly between groups (median, 10 mL [IQR, 0 to 48] vs. 6 mL [IQR, 0 to 24]; P = 0.088). Rates of intravenous thrombolysis and attempted thrombectomy were similar in the two groups (Table 1).

**Table 1:** Baseline demographic, clinical, and imaging characteristics of the study cohort (N = 253).

Variable	Overall	PVT-	PVT+	*P* value
	N = 253	N = 168	N = 85	
Age, Median (Q1, Q3)	67 (59, 78)	66 (55, 77)	70 (63, 81)	0.003
Sex, n (%)				0.009
Female	139 (55)	102 (61)	37 (44)	
Male	114 (45)	66 (39)	48 (56)	
Race, n (%)				0.39
Black	107 (42)	67 (40)	40 (47)	
White	126 (50)	89 (53)	37 (44)	
Asian	10 (4.0)	5 (3.0)	5 (5.9)	
Other/UTD	10 (4.0)	7 (4.2)	3 (3.5)	
Occlusion segment, n (%)				0.36
ICA	26 (10)	14 (8.3)	12 (14)	
M1	176 (70)	119 (71)	57 (67)	
M2	51 (20)	35 (21)	16 (19)	
Smoking status, n (%)	106 (42)	73 (43)	33 (39)	0.48
Hypertension, n (%)	199 (79)	129 (77)	70 (82)	0.31
Dyslipidemia, n (%)	134 (53)	90 (54)	44 (52)	0.79
Diabetes, n (%)	65 (26)	45 (27)	20 (24)	0.58
Atrial fibrillation, n (%)	95 (38)	58 (35)	37 (44)	0.16
History of Stroke/TIA, n (%)	49 (19)	32 (19)	17 (20)	0.86
Admission glucose level, Median (Q1, Q3)	119 (104, 144)	118 (103, 146)	121 (108, 140)	0.52
Admission NIHSS score, Median (Q1, Q3)	15 (10, 20)	13 (9, 20)	17 (13, 20)	0.003
Premorbid Modified rankin scale, Median (Q1, Q3)	0.00 (0.00, 1.00)	0.00 (0.00, 1.00)	0.00 (0.00, 2.00)	0.34
Cog-4, Median (Q1, Q3)	1.00 (0.00, 3.00)	0.00 (0.00, 2.50)	2.00 (0.00, 4.00)	<0.001
Stroke etiology (TOAST criteria), n (%)				0.63
Large artery atherosclerosis	41 (16)	29 (17)	12 (14)	
Cardioembolism	131 (52)	82 (49)	49 (58)	
Small vessel occlusion	0 (0)	0 (0)	0 (0)	
Stroke of other determined etiology	13 (5.2)	10 (6.0)	3 (3.5)	
Stroke of undetermined etiology	67 (27)	46 (28)	21 (25)	
Occlusion laterality, n (%)				0.89
left	125 (49)	84 (50)	41 (48)	
right	128 (51)	84 (50)	44 (52)	
ASPECTS, Median (Q1, Q3)	9.00 (7.00, 10.00)	9.00 (8.00, 10.00)	9.00 (7.00, 10.00)	0.41
rCBF <30% volume (mL), Median (Q1, Q3)	7 (0, 35)	6 (0, 24)	10 (0, 48)	0.088
Mismatch ratio, Median (Q1, Q3)	5 (3, 10)	5 (2, 9)	5 (3, 10)	0.49
CBV index <0.8, n (%)	105 (42)	68 (40)	37 (44)	0.64
HIR ≥ 0.4, n (%)	146 (58)	91 (54)	55 (65)	0.11
IVT administered, n (%)	86 (34)	58 (35)	28 (33)	0.8
MT attempted, n (%)	196 (77)	134 (80)	62 (73)	0.22
Modified thrombolysis in cerebral infarction (mTICI) score, n (%)				0.66
0	8 (4.1)	7 (5.3)	1 (1.6)	
1	5 (2.6)	4 (3.0)	1 (1.6)	
2a	8 (4.1)	7 (5.3)	1 (1.6)	
2b	45 (23)	31 (23)	14 (23)	
2c	28 (14)	17 (13)	11 (18)	
Type of thrombectomy, n (%)				0.93
Direct aspiration	99 (53)	68 (54)	31 (52)	
Stent retriever	17 (9.1)	12 (9.4)	5 (8.3)	
Combined	70 (37)	46 (36)	24 (40)	
Other	1 (0.5)	1 (0.8)	0 (0)	
Number of passes, Median (Q1, Q3)	1.00 (1.00, 2.00)	1.00 (1.00, 2.00)	1.00 (1.00, 2.00)	0.67
Type of anesthesia used, n (%)				0.2
General	184 (94)	128 (96)	56 (90)	
MAC	12 (6.1)	6 (4.5)	6 (9.7)	
FIV on DWI, Median (Q1, Q3)	27 (7, 80)	25 (7, 72)	30 (5, 127)	0.73
Admission to MRI (days), Median (Q1, Q3)	2.00 (1.00, 4.00)	2.00 (1.00, 4.00)	3.00 (1.00, 4.00)	0.17
Hemorrhagic transformation (HT), n (%)	89 (38)	58 (37)	31 (40)	0.65
PH, n (%)	30 (13)	16 (10)	14 (18)	0.089
Discharge NIHSS, Median (Q1, Q3)	5 (2, 12)	4 (1, 10)	7 (3, 14)	0.004
90-days mRS 0–2, n (%)	109 (53)	81 (61)	28 (39)	0.003
90-days mRS 0–1, n (%)	76 (37)	61 (46)	15 (21)	<0.001
90-days mRS 6 (Mortality), n (%)	35 (17)	17 (13)	18 (25)	0.023

### Discharge-stage higher cortical dysfunction (Cog-4)

Overall, the median discharge Cog-4 score was 1 (IQR, 0 to 3). Patients with PVT had higher (worse) Cog-4 scores than those without PVT (median, 2 [IQR, 0 to 4] vs. 0 [IQR, 0 to 2.5]; P < 0.001) (Table 1).

In univariable linear regression, PVT was associated with a higher discharge Cog-4 score (β, 1.1; 95% CI, 0.51 to 1.8; P < 0.001) (Table 2). In the multivariable model, PVT remained independently associated with higher discharge Cog-4 score (β, 0.63; 95% CI, 0.07 to 1.2; P = 0.029), after adjustment for age (β per year, 0.03; 95% CI, 0.01 to 0.05; P = 0.001), admission NIHSS score (β per point, 0.06; 95% CI, 0.02 to 0.11; P = 0.003), occlusion laterality (right vs. left: β, −1.8; 95% CI, −2.3 to −1.2; P < 0.001), and follow-up infarct volume on diffusion-weighted imaging (β per mL, 0.01; 95% CI, 0.00 to 0.01; P = 0.013) (Table 2). Baseline infarct core volume (rCBF <30% volume) was not independently associated with Cog-4 score in the adjusted model (P = 0.48) (Table 2).

**Table 2:** Univariable and Multivariable Linear Regression Analyses Examining the Association Between Clinical and Imaging Variables and Cog-4 Score at discharge.

Variable	Univariable models		Multivariable Model	
	Beta (95% CI)^a^	P	Beta (95% CI)^a^	p-value
Age	0.03 (0.01–0.05)	0.002	0.03 (0.01–0.05)	0.001
Sex				
Female	–			
Male	−0.04 (−0.66 to 0.58)	0.91		
Race				
Black	–			
White	−0.55 (−1.2 to 0.09)	0.090		
Asian	1.2 (−0.45 to 2.8)	0.16		
Other/UTD	−0.34 (−1.9 to 1.3)	0.68		
Occlusion segment				
ICA	–			
M1	−0.16 (−1.2 to 0.87)	0.76		
M2	−0.33 (−1.5 to 0.85)	0.58		
Smoking status	−0.26 (−0.89 to 0.36)	0.41		
Hypertension	0.62 (−0.13 to 1.4)	0.1		
Dyslipidemia	−0.45 (−1.1 to 0.17)	0.15		
Diabetes	0.49 (−0.21 to 1.2)	0.17		
Atrial fibrillation	0.30 (−0.34 to 0.94)	0.35		
History of Stroke/TIA	−0.15 (−0.93 to 0.63)	0.70		
Admission glucose level	0.00 (0.00–0.01)	0.35		
Admission NIHSS score	0.13 (0.09–0.17)	<0.001	0.06 (0.02–0.11)	0.003
Premorbid Modified rankin scale	0.15 (−0.13 to 0.43)	0.29		
Cog-4				
Stroke etiology (TOAST criteria)				
Large artery atherosclerosis	–			
Cardioembolism	0.24 (−0.65 to 1.1)	0.6		
Small vessel occlusion				
Stroke of other determined etiology	0.32 (−1.2 to 1.9)	0.69		
Stroke of undetermined etiology	0.03 (−0.94 to 1.0)	0.94		
Occlusion laterality				
left	–		–	
right	−1.8 (−2.4 to −1.3)	<0.001	−1.8 (−2.3 to −1.2)	<0.001
ASPECTS	−0.07 (−0.21 to 0.08)	0.37		
PVT	1.1 (0.51–1.8)	<0.001	0.63 (0.07–1.2)	0.029
rCBF <30% volume (mL)	0.02 (0.01–0.02)	0.002	0.00 (−0.01 to 0.02)	0.48
Mismatch ratio	−0.01 (−0.06 to 0.04)	0.70		
CBV index <0.8	0.40 (−0.22 to 1.0)	0.2		
HIR ≥ 0.4	0.36 (−0.27 to 0.98)	0.26		
IVT administered	−0.74 (−1.4 to −0.10)	0.024	−0.46 (−1.0 to 0.07)	0.091
MT attempted	−0.18 (−0.92 to 0.56)	0.64	−0.57 (−1.2 to 0.07)	0.081
FIV on DWI	0.01 (0.01–0.01)	<0.001	0.01 (0.00–0.01)	0.013

a CI = Confidence Interval.

### Discrimination for normal or minimal impairment at discharge

**Fig. 2:**
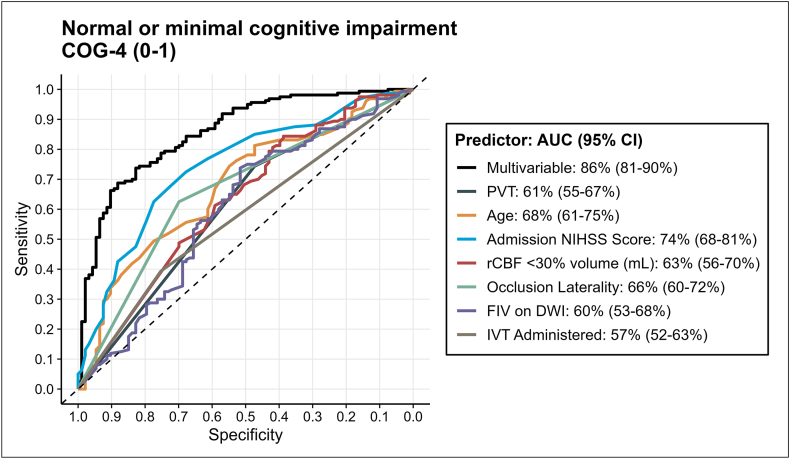
Discriminative performance of PVT and clinical predictors for identifying normal or minimal higher cortical deficits (Cog-4 score 0–1).

For identifying normal or minimal impairment (Cog-4 score, 0 to 1), the multivariable model showed an AUC of 0.86 (95% CI, 0.81 to 0.90) (Fig. 2). Discrimination for individual predictors was lower, including PVT (AUC, 0.61; 95% CI, 0.55 to 0.67), admission NIHSS score (AUC, 0.74; 95% CI, 0.68 to 0.81), and age (AUC, 0.68; 95% CI, 0.61 to 0.75) (Fig. 2).

### Functional outcomes

Patients with PVT had higher discharge NIHSS scores than those without PVT (median, 7 [IQR, 3 to 14] vs. 4 [IQR, 1 to 10]; P = 0.004) and were less likely to have functional independence at 90 days (mRS 0–2: 39% vs. 61%; P = 0.003) (Table 1). Mortality at 90 days was higher among patients with PVT than among those without PVT (25% vs. 13%; P = 0.023) (Table 1). In adjusted regression analysis of 90-day mRS shift, PVT was associated with worse outcome (β, 0.63; 95% CI, 0.07 to 1.2; P = 0.027) (Table 3).

**Table 3:** Univariable and Multivariable Linear Regression Analyses Examining the Association Between Clinical and Imaging Variables and modified Rankin shift.

Variable	Unadjusted Models		Adjusted Model	
	Beta (95% CI)^a^	P	Beta (95% CI)^a^	p-value
Age, Median (Q1, Q3)	0.02 (0.01–0.04)	0.01	0.03 (0.01–0.05)	<0.001
Sex, n (%)				
Female	–			
Male	0.15 (−0.47 to 0.76)	0.64		
Race, n (%)				
Black	–			
White	−0.74 (−1.4 to −0.11)	0.022		
Asian	−0.76 (−2.2 to 0.66)	0.29		
Other/UTD	−0.08 (−1.8 to 1.6)	0.93		
Occlusion segment, n (%)				
ICA	–			
M1	0.74 (−0.19 to 1.7)	0.12		
M2	0.39 (−0.70 to 1.5)	0.48		
Smoking status, n (%)	−0.08 (−0.69 to 0.53)	0.80		
Hypertension, n (%)	0.83 (0.10–1.6)	0.025		
Dyslipidemia, n (%)	−0.04 (−0.65 to 0.57)	0.90		
Diabetes, n (%)	0.56 (−0.12 to 1.2)	0.1		
Atrial fibrillation, n (%)	0.10 (−0.53 to 0.72)	0.76		
History of Stroke/TIA, n (%)	0.01 (−0.76 to 0.78)	0.98		
Admission glucose level, Median (Q1, Q3)	0.00 (0.00–0.01)	0.11		
Admission NIHSS score, Median (Q1, Q3)	0.08 (0.04–0.12)	<0.001	0.07 (0.02–0.11)	0.002
Premorbid Modified rankin scale, Median (Q1, Q3)	−0.55 (−0.82 to −0.29)	<0.001		
Stroke etiology (TOAST criteria), n (%)				
Large artery atherosclerosis	–			
Cardioembolism	0.51 (−0.36 to 1.4)	0.25		
Stroke of other determined etiology	0.67 (−1.1 to 2.5)	0.46		
Stroke of undetermined etiology	0.15 (−0.82 to 1.1)	0.76		
Occlusion laterality, n (%)				
left	–		–	
right	−0.78 (−1.4 to −0.19)	0.010	−1.8 (−2.3 to −1.2)	<0.001
ASPECTS, Median (Q1, Q3)	−0.11 (−0.25 to 0.02)	0.10		
rCBF <30% volume (mL), Median (Q1, Q3)	0.01 (0.01–0.02)	0.001	0.00 (−0.01 to 0.01)	0.54
PVT, n (%)	0.80 (0.17–1.4)	0.013	0.63 (0.07–1.2)	0.027
Mismatch volume (mL), Median (Q1, Q3)	0.00 (−0.01 to 0.00)	0.58		
IVT administered, n (%)	−0.72 (−1.4 to −0.09)	0.024	−0.47 (−1.0 to 0.07)	0.087
MT attempted, n (%)	0.25 (−0.46 to 0.96)	0.49	−0.61 (−1.3 to 0.06)	0.072
Modified thrombolysis in cerebral infarction (mTICI) score				
0	–			
1	−0.88 (−3.5 to 1.7)	0.51		
2a	−0.63 (−3.2 to 2.0)	0.63		
2b	−0.96 (−2.6 to 0.69)	0.25		
2c	−1.5 (−3.2 to 0.29)	0.10		
Symptom onset to door time (mins), Median (Q1, Q3)	0.00 (0.00–0.00)	0.73		
Door to CT time (minutes), Median (Q1, Q3)	0.00 (0.00–0.01)	0.31		
Door to needle time (minutes), Median (Q1, Q3)	0.00 (−0.01 to 0.02)	0.63		
Door to groin puncture time (minutes), Median (Q1, Q3)	0.00 (0.00–0.00)	0.24		
Groin puncture to first pass time (minutes), Median (Q1, Q3)	0.00 (0.00–0.00)	0.56		
Door to recanalization time (mins), Median (Q1, Q3)	0.00 (0.00–0.00)	0.85		
Groin puncture to recanalization time (minutes), Median (Q1, Q3)	0.00 (−0.01 to 0.02)	0.52		
FIV on DWI, Median (Q1, Q3)	0.01 (0.01–0.01)	<0.001	0.01 (0.00–0.01)	0.006

a CI = Confidence Interval.

### Sensitivity analysis

In a supplementary ordinal regression sensitivity analysis, PVT remained independently associated with higher discharge Cog-4 score, along with older age, higher admission NIHSS score, and left-sided occlusion (Supplementary Table 3).

## Discussion

In this cohort of 253 patients with anterior-circulation large-vessel occlusion, PVT on baseline perfusion imaging was independently associated with worse discharge-stage higher cortical dysfunction as captured by the NIHSS-derived cognitive items (Cog-4). Patients with PVT had higher discharge Cog-4 scores than those without PVT (median, 2 vs 0; P < 0.001). After adjustment for age, admission NIHSS score, occlusion laterality, and follow-up infarct volume on diffusion-weighted imaging, PVT remained associated with higher Cog-4 scores (β, 0.63; 95% CI: 0.07, 1.2; P = 0.029). A multivariable model incorporating these variables showed good discrimination for normal or minimal higher cortical deficits (Cog-4, 0–1) (AUC, 0.86; 95% CI: 0.81, 0.90), exceeding the performance of any single predictor, including PVT alone (AUC, 0.61; 95% CI: 0.55, 0.67) and admission NIHSS score (AUC, 0.74; 95% CI: 0.68, 0.81).

Our results support the emerging concept that the pathophysiology of AIS extends beyond the site of arterial occlusion and recanalization, with venous outflow functioning as a downstream driver of tissue injury and outcome [10,35,36]. Venous outflow profiles on CTA and perfusion imaging have repeatedly been linked to greater tissue edema, less favorable tissue-level reperfusion, and worse functional outcomes after thrombectomy, suggesting that venous physiology modulates how recanalized tissue evolves [[35], [36], [37], [38]]. Initial ischemia leads to cytotoxic and vasogenic edema, raising parenchymal and venous pressures, compressing thin-walled venules, and increasing resistance to venous drainage [39]. This phenomenon can trap deoxygenated blood in congested capillary beds, and sustain low-grade hypoperfusion in peri-infarct tissue, even when upstream arteries have been recanalized [11,40]. Such microvascular congestion and persistent tissue hypoperfusion provide a biologic rationale for why impaired venous outflow has been a strong predictor of global outcome in prior AIS-LVO series and, in our cohort, of discharge-stage higher cortical dysfunction [[30], [31], [32],^41^,^42^].

Building on this, there is growing evidence that venous congestion specifically affects cognitive function. In conditions characterized by chronic or subacute cerebral venous congestion, such as dural arteriovenous fistulas, cerebral venous sinus thrombosis, and broader cerebral venous congestion syndromes, formal testing frequently reveals impairments in attention, processing speed, and executive function that improve, often substantially, after venous decompression or fistula obliteration [[14], [15], [16],^43^,^44^]. Contemporary reviews conceptualize the cerebral venous system as a neurotoxic metabolites management network that not only returns blood but also participates in glymphatic and perivenous clearance of interstitial solutes via a glymphatic-venous axis [[45], [46], [47]]. Impaired venous outflow can disrupt these clearance pathways, leading to accumulation of neurotoxic metabolites, white matter injury, and progressive cognitive decline, as shown in experimental models and in human studies of cerebral small vessel disease and neurodegeneration [48,49]. Within this framework, PVT in AIS-LVO may represent an acute imaging marker of venous congestion and inefficient clearance, providing a mechanistic explanation for its independent association with worse discharge Cog-4 scores. Notably, PVT remained independently associated with worse discharge-stage higher cortical dysfunction even after adjustment for follow-up infarct volume on diffusion-weighted imaging, suggesting that venous outflow impairment conveys prognostic information that is not fully captured by final infarct size alone.

Beyond venous outflow, our findings are consistent with the broader post-stroke cognitive impairment literature regarding key clinical predictors. Older age was independently associated with higher Cog-4 scores, in line with large cohort and review data showing that age is one of the most robust determinants of both early and long-term post-stroke cognitive impairment [49]. The observed association between left-hemisphere occlusion and higher Cog-4 scores is also concordant with lesion-symptom mapping studies demonstrating that early cognitive impairment and MoCA abnormalities are predominantly driven by lesions in left frontal, temporal, and parietal networks, and with prior work showing that Cog-4 is biased toward detecting deficits in language-dominant hemispheric strokes [50].

PVT was also associated with worse functional outcome. Patients with PVT had lower rates of 90-day functional independence, higher mortality, and worse 90-day mRS shift after multivariable adjustment. These findings align with prior reports in successfully reperfused anterior-circulation large-vessel occlusion [37,51] and suggest that the prognostic significance of venous outflow impairment may not be limited to the successfully recanalized setting.

These findings provide a foundation for future work examining the relationship between venous outflow impairment and post-stroke cognitive outcomes. Assessment of PVT on routinely acquired perfusion maps is rapid and requires no additional imaging, making it feasible to incorporate into standard acute-stroke interpretation workflows alongside occlusion site, infarct core, and hypoperfusion burden. In the present study, PVT was associated with worse discharge-stage higher cortical dysfunction as captured by the NIHSS-derived cognitive items (Cog-4); however, Cog-4 is not a comprehensive cognitive assessment. Accordingly, prospective studies are needed to determine whether venous outflow impairment on admission predicts domain-specific cognitive impairment using standardized instruments such as the MoCA or MMSE and, when feasible, formal neuropsychological testing at prespecified follow-up intervals. If validated, venous outflow markers could complement existing imaging metrics to support early cognitive risk stratification, guide counseling of patients and caregivers, and inform referral for cognitive rehabilitation and structured outpatient cognitive follow-up.

This study has limitations. The retrospective and modest sample size limit generalizability and raise the possibility of residual confounding despite multivariable adjustment. Cognition was assessed with the NIHSS-derived cognitive items (Cog-4), which captures higher cortical deficits (language, neglect, and related items) rather than domain-specific cognition (e.g., memory or executive function). Accordingly, our findings should be interpreted as relating to discharge-stage higher cortical dysfunction rather than comprehensive post-stroke cognitive impairment. Prospective studies should validate PVT against standardized cognitive instruments (e.g., the MoCA) and, when feasible, domain-specific neuropsychological testing at prespecified follow-up time points [52,5,53]. Additionally, this study included only patients with both baseline perfusion imaging and a documented discharge Cog-4 score, which may introduce selection bias toward patients with intermediate stroke severity who survived to discharge with a complete NIHSS record; patients without perfusion imaging or with early death or transfer before discharge NIHSS were excluded, potentially limiting generalizability. Finally, imaging was acquired and postprocessed on a single perfusion platform and software package, which may affect the transportability of our specific PVT definition to other vendors and processing pipelines.

## Conclusion

PVT on baseline perfusion imaging was independently associated with worse discharge-stage higher cortical dysfunction as measured by the NIHSS-derived cognitive items (Cog-4) in patients with anterior-circulation large-vessel occlusion, even after accounting for follow-up infarct volume. These findings identify venous outflow impairment as a clinically relevant marker associated with higher cortical deficit burden early after stroke and provide a foundation for prospective studies using standardized cognitive assessments (e.g., MoCA or MMSE) and longitudinal follow-up to define implications for longer-term cognitive recovery.

## Author contributions

**Conceptualization:** Hamza Adel SalimVivek YedavalliMax WintermarkHamza Adel SalimVivek YedavalliMax WintermarkHamza Adel SalimAndrew ChoDerek TsangRisheng XuHamza Adel SalimAndrew ChoDerek TsangDhairya A. LakhaniRisheng XuShyam MajmundarMona GadVaibhav VagalShobit ChamoliKarthik LalwaniFerdinand HuiAdam A. DmytriwAdrien GuenegoKambiz NaelGregory W. AlbersJeremy J. HeitTobias D. FaizyVivek YedavalliMax WintermarkHamza Adel SalimHamza Adel SalimVivek YedavalliMax Wintermark

## Funding

None.

## Declaration of competing interest

The authors declare no competing interests.
