# Supplementary material for: Prolonged venous transit on perfusion imaging predicts discharge Cog-4 scores in anterior-circulation large-vessel occlusion stroke
Source: Neurotherapeutics. 2026 Jun 6;23(4):e00919. doi: 10.1016/j.neurot.2026.e00919 (PMC13264354; doi:10.1016/j.neurot.2026.e00919)
Supplement: Multimedia component 1 [file mmc1.docx]

Table 1: Univariable and Multivariable Linear Regression Analyses Examining the Association Between Clinical and Imaging Variables and modified Rankin shift

| **Variable** | **Unadjusted Models** | | **Adjusted Model** | |
| --- | --- | --- | --- | --- |
|  | **Beta** **(95% CI)***^2^* | **P** | **Beta** **(95% CI)***^2^* | **p-value** |
| Age, Median (Q1, Q3) | 0.02 (0.01 to 0.04) | 0.01 | 0.03 (0.01 to 0.05) | <0.001 |
| Sex, n (%) |  |  |  |  |
| Female | — |  |  |  |
| Male | 0.15 (-0.47 to 0.76) | 0.64 |  |  |
| Race, n (%) |  |  |  |  |
| Black | — |  |  |  |
| White | -0.74 (-1.4 to -0.11) | 0.022 |  |  |
| Asian | -0.76 (-2.2 to 0.66) | 0.29 |  |  |
| Other/UTD | -0.08 (-1.8 to 1.6) | 0.93 |  |  |
| Occlusion Segment, n (%) |  |  |  |  |
| ICA | — |  |  |  |
| M1 | 0.74 (-0.19 to 1.7) | 0.12 |  |  |
| M2 | 0.39 (-0.70 to 1.5) | 0.48 |  |  |
| Smoking Status, n (%) | -0.08 (-0.69 to 0.53) | 0.80 |  |  |
| Hypertension, n (%) | 0.83 (0.10 to 1.6) | 0.025 |  |  |
| Dyslipidemia, n (%) | -0.04 (-0.65 to 0.57) | 0.90 |  |  |
| Diabetes, n (%) | 0.56 (-0.12 to 1.2) | 0.1 |  |  |
| Atrial Fibrillation, n (%) | 0.10 (-0.53 to 0.72) | 0.76 |  |  |
| History of Stroke/TIA, n (%) | 0.01 (-0.76 to 0.78) | 0.98 |  |  |
| Admission Glucose Level, Median (Q1, Q3) | 0.00 (0.00 to 0.01) | 0.11 |  |  |
| Admission NIHSS Score, Median (Q1, Q3) | 0.08 (0.04 to 0.12) | <0.001 | 0.07 (0.02 to 0.11) | 0.002 |
| Premorbid Modified Rankin Scale, Median (Q1, Q3) | -0.55 (-0.82 to -0.29) | <0.001 |  |  |
| Stroke Etiology (TOAST Criteria), n (%) |  |  |  |  |
| Large artery atherosclerosis | — |  |  |  |
| Cardioembolism | 0.51 (-0.36 to 1.4) | 0.25 |  |  |
| Stroke of other determined etiology | 0.67 (-1.1 to 2.5) | 0.46 |  |  |
| Stroke of undetermined etiology | 0.15 (-0.82 to 1.1) | 0.76 |  |  |
| Occlusion Laterality, n (%) |  |  |  |  |
| left | — |  | — |  |
| right | -0.78 (-1.4 to -0.19) | 0.010 | -1.8 (-2.3 to -1.2) | <0.001 |
| ASPECTS, Median (Q1, Q3) | -0.11 (-0.25 to 0.02) | 0.10 |  |  |
| rCBF <30% volume (mL), Median (Q1, Q3) | 0.01 (0.01 to 0.02) | 0.001 | 0.00 (-0.01 to 0.01) | 0.54 |
| PVT, n (%) | 0.80 (0.17 to 1.4) | 0.013 | 0.63 (0.07 to 1.2) | 0.027 |
| Mismatch Volume (mL), Median (Q1, Q3) | 0.00 (-0.01 to 0.00) | 0.58 |  |  |
| IVT Administered, n (%) | -0.72 (-1.4 to -0.09) | 0.024 | -0.47 (-1.0 to 0.07) | 0.087 |
| MT Attempted, n (%) | 0.25 (-0.46 to 0.96) | 0.49 | -0.61 (-1.3 to 0.06) | 0.072 |
| Symptom Onset to Door Time (mins), Median (Q1, Q3) | 0.00 (0.00 to 0.00) | 0.73 |  |  |
| Door to CT Time (minutes), Median (Q1, Q3) | 0.00 (0.00 to 0.01) | 0.31 |  |  |
| Door to Needle Time (minutes), Median (Q1, Q3) | 0.00 (-0.01 to 0.02) | 0.63 |  |  |
| Door to Groin Puncture Time (minutes), Median (Q1, Q3) | 0.00 (0.00 to 0.00) | 0.24 |  |  |
| Groin Puncture to First Pass Time (minutes), Median (Q1, Q3) | 0.00 (0.00 to 0.00) | 0.56 |  |  |
| Door to Recanalization Time (mins), Median (Q1, Q3) | 0.00 (0.00 to 0.00) | 0.85 |  |  |
| Groin Puncture to Recanalization Time (minutes), Median (Q1, Q3) | 0.00 (-0.01 to 0.02) | 0.52 |  |  |
| FIV on FLAIR, Median (Q1, Q3) | 0.00 (0.00 to 0.00) | 0.16 |  |  |
| FIV on DWI, Median (Q1, Q3) | 0.01 (0.01 to 0.01) | <0.001 | 0.01 (0.00 to 0.01) | 0.006 |
| *^2^* CI = Confidence Interval | | | | |

Supplementary Table 2. Univariable and multivariable ordinal regression analyses of factors associated with Cog-4 score

| **Variable** | **Univariable models** | | **Multivariable Model** | |
| --- | --- | --- | --- | --- |
|  | **OR (95% CI)***^2^* | **P** | **OR (95% CI)***^2^* | **p-value** |
| Age, Median (Q1, Q3) | 1.03 (1.01 to 1.04) | <0.001 | 1.03 (1.02 to 1.05) | <0.001 |
| Sex, n (%) |  |  |  |  |
| Female | — |  |  |  |
| Male | 1.02 (0.65 to 1.61) | 0.92 |  |  |
| Race, n (%) |  |  |  |  |
| Black | — |  |  |  |
| White | 0.64 (0.40 to 1.03) | 0.068 |  |  |
| Asian | 2.21 (0.68 to 7.09) | 0.18 |  |  |
| Other/UTD | 1.22 (0.43 to 3.42) | 0.7 |  |  |
| Occlusion Segment, n (%) |  |  |  |  |
| ICA | — |  |  |  |
| M1 | 0.85 (0.41 to 1.83) | 0.68 |  |  |
| M2 | 0.84 (0.36 to 1.99) | 0.69 |  |  |
| Smoking Status, n (%) | 0.78 (0.49 to 1.24) | 0.3 |  |  |
| Hypertension, n (%) | 1.92 (1.08 to 3.50) | 0.028 |  |  |
| Dyslipidemia, n (%) | 0.87 (0.55 to 1.37) | 0.54 |  |  |
| Diabetes, n (%) | 1.55 (0.93 to 2.57) | 0.091 |  |  |
| Atrial Fibrillation, n (%) | 1.21 (0.76 to 1.93) | 0.42 |  |  |
| History of Stroke/TIA, n (%) | 0.93 (0.52 to 1.63) | 0.79 |  |  |
| Admission Glucose Level, Median (Q1, Q3) | 1.00 (1.00 to 1.01) | 0.15 |  |  |
| Admission NIHSS Score, Median (Q1, Q3) | 1.12 (1.08 to 1.16) | <0.001 | 1.07 (1.02 to 1.11) | 0.002 |
| Premorbid Modified Rankin Scale, Median (Q1, Q3) | 1.15 (0.94 to 1.40) | 0.18 |  |  |
| Cog-4, Median (Q1, Q3) |  |  |  |  |
| Stroke Etiology (TOAST Criteria), n (%) |  |  |  |  |
| Large artery atherosclerosis | — |  |  |  |
| Cardioembolism | 1.18 (0.63 to 2.25) | 0.61 |  |  |
| Small vessel occlusion |  |  |  |  |
| Stroke of other determined etiology | 0.97 (0.28 to 3.17) | 0.96 |  |  |
| Stroke of undetermined etiology | 0.92 (0.45 to 1.88) | 0.81 |  |  |
| Occlusion Laterality, n (%) |  |  |  |  |
| left | — |  | — |  |
| right | 0.28 (0.18 to 0.45) | <0.001 | 0.20 (0.12 to 0.35) | <0.001 |
| ASPECTS, Median (Q1, Q3) | 0.95 (0.86 to 1.05) | 0.33 |  |  |
| PVT, n (%) | 2.44 (1.52 to 3.95) | <0.001 | 1.85 (1.11 to 3.08) | 0.018 |
| rCBF <30% volume (mL), Median (Q1, Q3) | 1.01 (1.01 to 1.02) | <0.001 | 1.01 (1.00 to 1.02) | 0.17 |
| Mismatch Ratio, Median (Q1, Q3) | 1.00 (0.96 to 1.03) | 0.83 |  |  |
| CBV index < 0.8, n (%) |  |  |  |  |
| HIR>=0.4, n (%) |  |  |  |  |
| IVT Administered, n (%) | 0.58 (0.36 to 0.94) | 0.028 | 0.65 (0.39 to 1.11) | 0.11 |
| MT Attempted, n (%) | 1.05 (0.60 to 1.83) | 0.87 | 0.58 (0.31 to 1.09) | 0.091 |
| FIV on FLAIR, Median (Q1, Q3) | 1.00 (1.00 to 1.00) | 0.59 |  |  |
| FIV on DWI, Median (Q1, Q3) | 1.01 (1.00 to 1.01) | <0.001 | 1.00 (1.00 to 1.01) | 0.33 |
| *^2^* CI = Confidence Interval | | | | |
